# Impact of Thrombocytopenia on In-Hospital Outcome in Patients Undergoing Percutaneous Coronary Intervention

**DOI:** 10.1155/2021/8836450

**Published:** 2021-01-13

**Authors:** Zhongxiu Chen, Zheng Liu, Nan Li, Ran Liu, Miye Wang, Duolao Wang, Chen Li, Kai Li, Fangbo Luo, Yong He

**Affiliations:** ^1^Department of Cardiology, West China Hospital of Sichuan University, Chengdu, Sichuan, China; ^2^Department of Nursing, West China Hospital of Sichuan University, Chengdu, Sichuan, China; ^3^Information Center of West China Hospital of Sichuan University, Chengdu, Sichuan, China; ^4^Department of Clinical Sciences, Liverpool School of Tropical Medicine, Pembroke Place, Liverpool L3 5QA, UK; ^5^Department of Rehabilitation, Community Health Center of Huaxing Wuhou District, Chengdu, Sichuan, China

## Abstract

**Background:**

Thrombocytopenia was intuitively considered to be associated with higher risk of bleeding and multiple comorbidities after percutaneous coronary intervention (PCI). However, controversial results exist, and the real-world clinical impact of thrombocytopenia in patients undergoing PCI is largely unknown. The aim of this study was to evaluate the influence of baseline thrombocytopenia on the prognosis of patients undergoing PCI.

**Methods:**

Using the West China Hospital Inpatient Sample database, patients who underwent PCI were identified from August 2012 to January 2019. Baseline thrombocytopenia was defined as a preprocedural platelet count of 100 × 10^9^/L or less obtained from a routine blood sample taken within 48 hours before coronary PCI. The clinical effect of the advanced thrombocytopenia group (≤85 × 10^9^/L), according to the median value of platelet count in the thrombocytopenia cohort, was further assessed. The primary outcome was a composite of in-hospital death, bleeding events, and post-PCI transfusion.

**Results:**

Of 9531 patients enrolled in our study, 936 had baseline thrombocytopenia and 8595 patients did not have. There were no significant differences in the primary outcome between the two groups. However, advanced thrombocytopenia was independently associated with higher risk of primary outcome (OR 1.67, 95% CI 1.06 to 2.65, *p* = 0.029). Acute coronary syndrome (ACS) patients with thrombocytopenia were associated with higher odds of major bleeding (BARC ≥ 2) (OR 2.56, 95% CI 1.24 to 5.44, *p* = 0.011). Compared with the nonthrombocytopenia group, the thrombocytopenia group with ticagrelor use had higher odds of major bleeding (OR 9.7, 95% CI 1.57 to 60.4 versus OR 0.22, 95% CI 0.03 to 1.69, interaction *p* = 0.025).

**Conclusions:**

It seems feasible for patients with thrombocytopenia to receive PCI, but close attention should be paid to advanced thrombocytopenia, the risk of postprocedure bleeding in ACS patients, and the use of more potent P2Y_12_ inhibitor.

## 1. Introduction

The management of antiplatelet therapy/percutaneous coronary intervention (PCI) in coronary artery disease (CAD) patients with thrombocytopenia poses a particular challenge for physicians, as they are at higher risk of both bleeding and, paradoxically, ischemic events [1]. The reported incidence of baseline thrombocytopenia was varied approximately from 1% to 13% of CAD patients [2, 3]. However, these patients have almost invariably been excluded from randomized clinical trials of PCI or antithrombotic therapies [4, 5]. No guidelines and only several reports are available on the safety and tolerance of antiplatelet therapy/PCI in CAD patients complicated with thrombocytopenia at baseline, and the results are still controversial. Previous studies revealed that patients with baseline thrombocytopenia had higher risk for bleeding events and death [3, 6], while no differences in mortality or bleeding complications between PCI patients with and without thrombocytopenia were also demonstrated [2, 7, 8]. Hence, the real impact of thrombocytopenia in patients undergoing PCI is largely unknown. Therefore, this large cohort study was aimed at evaluating the effect of baseline thrombocytopenia on the prognosis of patients undergoing PCI according to the current practice.

## 2. Materials and Methods

### 2.1. Study Design and Subjects

**Figure 1:**
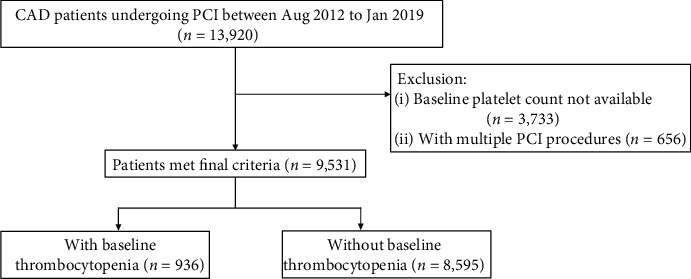
Study flowchart describing inclusion and exclusion criteria leading to the final cohort of patients. CAD: coronary artery disease; PCI: percutaneous coronary intervention.

This retrospective cohort study included 13,920 consecutively enrolled CAD patients with PCI from August 1, 2012, to January 30, 2019, at West China Hospital. The study protocol was approved by the Ethics Committee of West China Hospital of Sichuan University (Sichuan, China). 3733 patients without complete blood count analysis before PCI were excluded. For patients with multiple PCI procedures, only the first PCI in the time period was included, leaving 9531 patients for analysis (Figure 1). For the purpose of this study, baseline thrombocytopenia was defined as a preprocedural platelet count of 100 × 10^9^/L or less obtained from a routine blood sample taken within 48 hours before coronary PCI. The thrombocytopenia cohort was further classified into patients with intermediate thrombocytopenia (85 × 10^9^/L < platelet count ≤ 100 × 10^9^/L) and advanced thrombocytopenia (≤85 × 10^9^/L) according to the median value of platelet count of this thrombocytopenia group.

### 2.2. Data Collection

Clinical data were extracted from the Hospital Information System and obtained through a review of medical records. Baseline characteristics, medical history, comorbidities, and in-hospital management based on prescription records were evaluated by experienced cardiologists. Laboratory test results of enrolled subjects performed in the West China Hospital Biochemistry Laboratory were also collected.

### 2.3. Clinical Outcomes

The primary outcome was a composite of in-hospital all-cause death, bleeding events, and post-PCI transfusion. Secondary outcomes included major adverse cardiovascular events (MACE); a composite of in-hospital cardiac mortality, myocardial infarction (MI), target vessel revascularization (TVR), or stent thrombosis; ischemic and hemorrhagic cerebrovascular accident (CVA); and length of stay. The Bleeding Academic Research Consortium (BARC) definition for bleeding was used to describe bleeding events [9]. Major bleeding was categorized as type 2, 3, or 5 (type 2 indicating any overt, actionable sign of bleeding; type 3 indicating bleeding with a decrease in the hemoglobin of >3 g per deciliter, any transfusion, cardiac tamponade, or intracranial or ocular involvement; and type 5 indicating fatal bleeding). The MACE endpoints were defined in accordance with the Academic Research Consortium (ARC) definitions [10]. MI was defined according to the third Universal Definition of MI [11]. For PCI-related MI, we used higher biomarker thresholds for confirmation according to the ISCHEMIA trial (International Study of Comparative Health Effectiveness with Medical and Invasive Approaches) [12]. Cardiac mortality was defined as a documented arrhythmogenic death, an unexpected presumed pulseless condition with the absence of an obvious noncardiac explanation, or death due to congestive cardiac failure or structural heart disease. CVA was defined as an acute episode of focal or global neurological dysfunction persisting for more than 24 hours or leaving residual signs and confirmed by skull CT or MRI. Hospital records were searched for all keywords associated with potential bleeding events, including bleeding and transfusion, and a review of medical records. Outcomes were interpreted and judged by an independent cardiologist.

### 2.4. Statistical Analysis

There were no missing data for the included variables. Some variables with missing data, such as estimated glomerular filtration rate and left ventricular ejection fraction, were not included in our study. All analyses were performed with SPSS version 23 (IBM Corporation, Armonk, NY). Continuous variables were expressed as mean ± standard deviation or median with 25^th^ and 75^th^ interquartile range and were compared using the Student *t*-test or Wilcoxon rank-sum test depending on their distributions. Categorical variables were presented as number and percentage and were compared with the *χ*^2^ or Fisher exact test. Differences in outcomes were reported as odds ratios (OR) with 95% confidence intervals (CI). To address the possible imbalance in the participants' characteristics between the test groups, a propensity score approach was used. The propensity score predicting thrombocytopenia was generated using multivariable logistic regression with thrombocytopenia as the dependent variable and fifteen prespecified baseline characteristics and traditional risk factors (age, sex, hypertension, diabetes mellitus, atrial fibrillation, hyperlipidemia, congestive heart failure, chronic kidney disease, chronic liver disease, prior cerebrovascular accident, prior PCI, prior CABG, stents placed (≥2), use of intra-aortic balloon pump, and arterial approach) as the independent variables. Logistic regression was performed to identify the association between thrombocytopenia and the risk of clinical outcomes. We used the propensity score as a control variable in the covariate-adjusted analysis of the outcome variables [13]. The association between thrombocytopenia and lg10-transformed length of stay (due to lack of normality) was assessed by linear regression analysis. All reported *p* values were 2-tailed, and *p* values < 0.05 were considered statistically significant.

## 3. Results

### 3.1. Baseline Characteristics

Of 9531 PCI procedures, 936 patients (9.8%) had thrombocytopenia at baseline. The median platelet count in patients with thrombocytopenia was 85 × 10^9^/L (25th, 75th percentiles, 74 × 10^9^/L, 94 × 10^9^/L; range, 6 to 100 × 10^9^/L). All PCI patients used a second-generation drug-eluting stent, and approximately 95% of the stent type was Promus PREMIER™ (MONORAIL™, Boston Scientific, USA) and 5% was GuReater® (CoCr Sirolimus-Eluting Coronary Stent System, LEPU MEDICAL, China). Compared with patients without thrombocytopenia, patients with thrombocytopenia were older, were more commonly men, had higher level of creatinine and total bilirubin, and had a higher prevalence of atrial fibrillation, chronic kidney disease, and chronic liver disease and lesser use of aspirin (Table 1).

### 3.2. In-Hospital Clinical Outcomes

Compared with PCI patients without thrombocytopenia, patients with thrombocytopenia had higher prevalence of primary outcome (4.0% vs. 2.5%, *p* = 0.009), including higher prevalence of major bleeding (BARC ≥ 2) (1.3% vs. 0.6%, *p* = 0.014) and transfusion (1.9% vs. 1.0%, *p* = 0.016). No significant difference in the secondary outcome was detected between the groups, while the thrombocytopenia cohort had higher prevalence of TVR (0.3% vs. 0.1%, *p* = 0.042). Patients with thrombocytopenia had longer length of hospital stay (5 (3–7) days vs. 4 (3–6) days, *p* = 0.007). After adjusting for the propensity scores in a logistic or linear regression model, thrombocytopenia showed no effects on the rates of primary and secondary outcomes except that thrombocytopenia was associated with higher risk of TVR (OR 4.93, 95% CI 1.13 to 21.46, *p* = 0.03) (Table 2). Additionally, compared with PCI patients with intermediate thrombocytopenia, the rate of primary outcome, including major bleeding (BARC ≥ 2) and transfusion, was significantly higher in the PCI cohort with advanced thrombocytopenia (Supplemental Table 1). After adjusting for the propensity scores in a logistic regression model, advanced thrombocytopenia was associated with higher risk of primary outcome (OR 1.67, 95% CI 1.06 to 2.65, *p* = 0.029), including higher risk of major bleeding (OR 2.35, 95% CI 1.11 to 5.00, *p* = 0.027) and transfusion (OR 2.16, 95% CI 1.17 to 4.00, *p* = 0.014) (Table 3).

### 3.3. Clinical Outcomes in Patients with Acute Coronary Syndrome

Compared with acute coronary syndrome (ACS) patients without thrombocytopenia, ACS patients with thrombocytopenia had higher prevalence of primary outcome (5.4% vs. 3.1%, *p* = 0.007), including higher prevalence of all-cause mortality (2.6% vs. 1.3%, 8), bleeding (2.4% vs. 1.2%, *p* = 0.024), major bleeding (BARC ≥ 2) (2.2% vs. 0.7%, *p* = 0.001), and transfusion (2.6% vs. 1.4%, *p* = 0.027). No significant difference in the secondary outcome was detected between the groups (Table 4). After adjusting for the propensity scores, ACS patients with thrombocytopenia were only associated with higher risk of major bleeding (BARC ≥ 2) (OR 2.56, 95% CI 1.24 to 5.44, *p* = 0.011), while there were no effects on the rates of other in-hospital outcomes. There were no significant differences in clinical outcomes in the chronic coronary syndrome cohort with or without thrombocytopenia (Supplemental Table 2).

### 3.4. Clinical Outcomes Stratified by Usage of Ticagrelor

**Figure 2:**
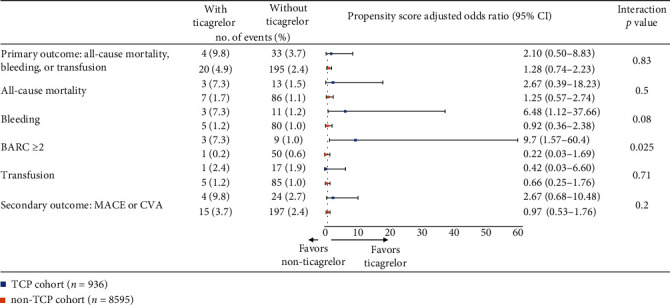
The association between ticagrelor use and the risk of in-hospital outcomes in patients with and without TCP. TCP: thrombocytopenia; BARC: Bleeding Academic Research Consortium; MACE: major adverse cardiovascular events; CVA: cerebrovascular accident.

Specifically, among patients who received more potent P2Y_12_ inhibitor (ticagrelor), we determined if there was variability in ticagrelor-associated outcomes in PCI patients with thrombocytopenia. As shown in Figure 2, there were no significant differences in the treatment effect of ticagrelor in rates of primary outcome in patients with thrombocytopenia (OR 2.10, 95% CI 0.50 to 8.83) or without thrombocytopenia (OR 1.28, 95% CI 0.74 to 2.23, interaction *p* = 0.83). Likewise, no statistical evidence of heterogeneity was found between the thrombocytopenia and nonthrombocytopenia cohorts for the secondary outcome. However, compared with the nonthrombocytopenia group, we did observe higher odds of bleeding (OR 6.48, 95% CI 1.12 to 37.66 versus OR 0.92, 95% CI 0.36 to 2.38, interaction *p* = 0.08) and major bleeding (OR 9.7, 95% CI 1.57 to 60.4 versus OR 0.22, 95% CI 0.03 to 1.69, interaction *p* = 0.025) in the thrombocytopenia group with ticagrelor use.

## 4. Discussion

Several important findings were observed in this large retrospective study of contemporary domestic real-world clinical practices of PCI and periprocedural antithrombotic use among 9531 CAD patients from over approximately 8 years from 2012 to 2019. Firstly, the incidence of baseline thrombocytopenia in patients undergoing PCI was common, occurring in approximately one in ten patients. Secondly, although there is high prevalence of baseline thrombocytopenia, the incidence of in-hospital adverse events did not increase significantly in the thrombocytopenia group when compared with the normal platelet group. However, advanced thrombocytopenia was independently associated with higher risk of the primary outcome. Thirdly, thrombocytopenia in the ACS subgroup was associated with higher risk of in-hospital major bleeding, while there were no effects on the rates of all-cause mortality, cardiac mortality, CVA, and transfusion and on the length of hospital stay. Fourthly, higher risks of bleeding or major bleeding were observed when ticagrelor was used in thrombocytopenia patients.

Our results are consistent with Raphael et al.'s [8] and Liu et al.'s [2] findings. In Raphael et al.'s retrospective analysis (thrombocytopenia was defined as platelet count < 100 × 10^9^/L, *n* = 204), in-hospital bleeding events and death after PCI were similar in patients with and without thrombocytopenia. Although a higher number of bleeding events in the thrombocytopenia group were detected on follow-up, this phenomenon happened at 5 years after PCI and was largely gastrointestinal in nature, unrelated to the PCI procedure and dual antiplatelet therapy. Liu et al.'s study was aimed at investigating the long-term impact of thrombocytopenia (defined as platelet count < 150 × 10^9^/L, *n* = 1263) in Chinese patients undergoing elective PCI. No differences in 30-month adverse outcomes, including mortality and bleeding complications, were detected between patients with and without thrombocytopenia. Additionally, in cancer patients with ACS and chronic thrombocytopenia, no procedure- or antiplatelet therapy-related cerebrovascular events were noted. Moderate thrombocytopenia was associated with decreased overall survival, whereas aspirin, dual antiplatelet therapy, and statin use showed a trend of improved overall survival [14]. These results indicated that it seemed feasible for patients with moderate chronic thrombocytopenia to receive PCI as well as guideline-recommended duration of antiplatelet therapy.

Controversially, some studies demonstrated opposite conclusion. Ito et al. [3] evaluated the influence of thrombocytopenia (defined as platelet count < 150 × 10^9^/L) on PCI patients in the pooled database from 3 prospective studies in Japan, CREDO- (Coronary Revascularization Demonstrating Outcome-) Kyoto PCI/CABG registry cohort-2, RESET (the Randomized Evaluation of Sirolimus-Eluting Versus Everolimus-Eluting Stent Trial), and NEXT (NOBORI Biolimus-Eluting Versus XIENCE/PROMUS Everolimus-Eluting Stent Trial), and demonstrated that even mild thrombocytopenia at baseline was associated with higher risk for bleeding events and all-cause death through a 3-year follow-up (both within and beyond 30 days). Yadav et al. [15] reported that baseline thrombocytopenia was not associated with the crude event at 30 days but represented a hematologic marker of poor prognosis and was strongly associated with adverse ischemic events 1 year after PCI in pooled populations from 2 large-scale randomized trials: the ACUITY (Acute Catheterization and Urgent Intervention Triage Strategy) [16] and HORIZONS-AMI (Harmonizing Outcomes With Revascularization and Stents in Acute Myocardial Infarction) [17] trials. More recently, Ayoub et al.'s study [6] identified patients who underwent PCI with or without chronic thrombocytopenia from the National Inpatient Sample database of the UK and revealed that patients with chronic thrombocytopenia were at higher risk of in-hospital mortality (OR 2.30, 95% CI 1.90 to 2.70, *p* < 0.0001) and bleeding complications (OR 2.40, 95% CI 2.05 to 2.72, *p* < 0.0001).

The contrary scenario can be attributed to the different cut-off values of platelet count for thrombocytopenia, study population, disease severity, definitions of bleeding criteria, PCI strategy and antithrombotic regimen, the exclusion of patients with severe thrombocytopenia from some studies, and varying follow-up durations. For example, in Ito et al.'s pooled study of 3 different era researches [3], bare-metal stent, the first-generation DES, or older antiplatelet agents (ticlopidine) were used in a large proportion of patients (different from the current PCI practice), and bleeding was defined in the Global Utilization of Streptokinase and Tissue Plasminogen Activator for Occluded arteries trial (GUSTO). ACUITY and HORIZONS-AMI trials recruited patients with acute coronary syndromes, excluded all patients with platelet counts < 100 × 10^9^/L, had more severe disease in the thrombocytopenia group (100-150 × 10^9^/L), and routinely used glycoprotein IIb/IIIa inhibitor, which is selective for bailout use according to the current practice. In our cohort, we did observe higher bleeding risk with usage of tirofiban in both thrombocytopenia and nonthrombocytopenia cohorts, while it is more obvious in the thrombocytopenia cohort (Supplemental Figure 1), which furtherly confirmed the higher bleeding effect of the combined use of glycoprotein IIb/IIIa inhibitor.

In Ayoub et al.'s [6] large cohort study, chronic thrombocytopenia patients (durations of >1 year) were identified by administrative code at discharge from a claim-based database, not validated by single patients' blood counts. Thus, the degree of thrombocytopenia might be more severe and more caused by malignancy or chronic liver disease and associated with poorer outcomes. Moreover, studies reported that the association between platelet count and long-term mortality functioned in a U-shaped fashion [18], with both lower and higher platelet counts associated with a greater mortality risk. Ito et al.'s study [3] confirmed the U-shaped model. The retrospective analysis from the Mayo Clinic revealed that a platelet count of less than 50 × 10^9^/L was considered more significantly increased bleeding risk [8]. Consistently, in our study, after adjusting for the propensity scores in a logistic regression model, we found that advanced thrombocytopenia was associated with higher risk of the primary outcome. Thus, special attention should be paid to PCI patients with advanced/severe thrombocytopenia.

Recently, the Academic Research Consortium for High Bleeding Risk (ARC-HBR) reported a standardized definition of HBR (a predicted annual BARC 3 or 5 bleeding rate of ≥4% or an intracranial hemorrhage risk of ≥1%) in patients undergoing PCI [19]. Anticipated use of long-term oral anticoagulation, severe or end-stage chronic kidney disease (eGFR < 30 mL/min), hemoglobin < 11 g/dL, moderate or severe baseline thrombocytopenia (platelet count < 100 × 109/L), and chronic bleeding diathesis are considered important factors of HBR. Moreover, low body weight, frailty, heart failure, and dialysis are listed as risk factors of the HBR criteria for Asian patients [20]. Our study showed that baseline thrombocytopenia did not appear to have a clinically significant effect on in-hospital adverse outcomes among patients who underwent PCI, while the risk of bleeding was increased in the ACS subgroup with thrombocytopenia and in thrombocytopenia patients with ticagrelor. Given the demonstrated link between bleeding and increased mortality after PCI [21, 22], it is important for clinicians to pay close attention to the risk of postprocedure bleeding in ACS patients and cautiously use ticagrelor because of its higher bleeding risk [1, 23, 24]. In our study, a total of 3 patients had hemorrhagic CVA and all were in the nonthrombocytopenia cohort. That means, besides platelet number, clinicians should also pay attention to the function of platelets and take other bleeding risk factors, such as other major and minor HBR criteria, into account comprehensively, and recommended methods for minimizing bleeding risk, including favoring the radial access, careful dosing of antithrombotic agents, and prescribing a proton pump inhibitor, should be taken in high bleeding risk patients. Similar to Eikelboom et al.'s [25] and Yadav et al.'s [15] observation, other used antithrombotic agent did not have a significant effect on the association between baseline thrombocytopenia and in-hospital adverse clinical events in our study. This reminds us that baseline thrombocytopenia is most probably not a mediator of mortality in most cases, and less intensive antithrombotic during or after PCI in patients with baseline thrombocytopenia may lead to the increase in ischemic events. The higher odds of TVR in the thrombocytopenia group of our study may be attributed to its lesser periprocedural prescription of aspirin. Therefore, we should assess comprehensively the severity of the atherothrombotic event and the overall clinical status for prognosis prediction.

### 4.1. Limitations

This study has several limitations. Firstly, despite the use of the propensity score approach for controlling for possible confounders, the observed results may still subject to imbalances between patients with or without thrombocytopenia due to unmeasured confounders, such as clotting of platelet when collecting blood samples occurring sometimes in the clinical settings, which may mislead to thrombocytopenia. Secondly, the causes of baseline thrombocytopenia were not investigated in every case, and heparin or antiplatelet drug administration prehospital could potentially influence platelet counts. However, the hazard of thrombocytopenia was adjusted with the presentation of acute myocardial infarction and previous PCI, CABG, and stroke, which is linked with antiplatelet therapy. Thirdly, the sample of PCI patients with ticagrelor was relatively small because ticagrelor only became available in China in recent years. Prospective randomized studies are needed to provide robust data. Lastly, we did not explore the long-term effect of baseline thrombocytopenia, and only nine patients had severe thrombocytopenia in our study. Thus, future prospective studies with larger sample size and more patients with severe thrombocytopenia are required.

## 5. Conclusions

Although baseline thrombocytopenia was common among patients who underwent PCI, it did not appear to have a clinically significant effect on in-hospital adverse outcomes. However, adverse events were increased in patients with advanced thrombocytopenia. In addition, bleeding risk was increased in the ACS subgroup with thrombocytopenia and was significantly higher in thrombocytopenia patients with ticagrelor. This retrospective single-center study indicated that it seemed feasible for patients with intermediate thrombocytopenia to receive PCI as well as conventional antiplatelet therapy. Clinicians should pay close attention to the advanced thrombocytopenia cohort and the risk of postprocedure bleeding in ACS patients and cautiously use potent P2Y_12_ inhibitor, such as ticagrelor.

## Figures and Tables

**Table 1:** Demographic and clinical characteristics of patients with and without thrombocytopenia.

Variable	With TCP (*n* = 936)	Without TCP (*n* = 8595)	*p* value
Mean age (yrs)	67.1 ± 10.2	64.6 ± 11.5	<0.001
Male, *n* (%)	758 (81.0)	6614 (77.0)	0.005
Platelet (×10^9^/L)	85 (74–94)	174 (141–215)	<0.001
Creatinine (*μ*mol/L)	85 (72–103)	81 (69–95)	<0.001
Total bilirubin (*μ*mol/L)	13.1 (9.8–17.2)	10.9 (8.0–14.8)	<0.001
Hypertension, *n* (%)	514 (54.9)	4949 (57.6)	0.12
Diabetes mellitus, *n* (%)	273 (29.2)	2409 (28.0)	0.46
Atrial fibrillation, *n* (%)	54 (5.8)	332 (3.9)	0.005
Hyperlipidemia, *n* (%)	68 (7.2)	714 (8.3)	0.27
Chronic kidney disease, *n* (%)	55 (5.9)	316 (3.7)	0.001
Chronic liver disease, *n* (%)	18 (1.9)	44 (0.5)	<0.001
Unstable angina, *n* (%)	265 (28.3)	2330 (27.1)	0.43
NSTEMI, *n* (%)	92 (9.8)	813 (9.5)	0.71
STEMI, *n* (%)	140 (15.0)	1445 (16.8)	0.15
Cardiogenic shock, *n* (%)	23 (2.5)	145 (1.7)	0.09
Congestive heart failure, *n* (%)	112 (12.0)	856 (10.0)	0.054
Prior cerebrovascular accident, *n* (%)	21 (2.2)	234 (2.8)	0.39
Prior PCI, *n* (%)	125 (13.4)	1124 (13.1)	0.81
Prior CABG, *n* (%)	8 (0.9)	53 (0.6)	0.39
IABP, *n* (%)	53 (5.7)	397 (4.6)	0.15
Stents placed (≥2), *n* (%)	278 (29.7)	2329 (27.1)	0.09
Arterial approach			0.27
Radial, *n* (%)	843 (90.1)	7844 (91.3)	
Femoral, *n* (%)	47 (5.0)	407 (4.7)	
Both, *n* (%)	36 (3.8)	237 (2.8)	
Other, *n* (%)	10 (1.1)	107 (1.2)	
Periprocedural medicine			
Aspirin, *n* (%)	903 (96.5)	8449 (98.3)	<0.001
Clopidogrel, *n* (%)	907 (96.9)	8321 (96.8)	0.88
Ticagrelor, *n* (%)	41 (4.4)	410 (4.8)	0.59
Warfarin, *n* (%)	12 (1.3)	116 (1.3)	0.87
NOAC, *n* (%)	3 (0.3)	27 (0.3)	1.0
Tirofiban, *n* (%)	161 (17.2)	1531 (17.8)	0.64
Enoxaparin, *n* (%)	254 (27.1)	2448 (28.5)	0.39

TCP: thrombocytopenia; LDL-C: low-density lipoprotein cholesterol; NSTEMI: non-ST-segment elevation myocardial infarction; STEMI: ST-segment elevation myocardial infarction; PCI: percutaneous coronary intervention; CABG: coronary artery bypass graft; IABP: intra-aortic balloon pump; NOAC: new oral anticoagulants.

**Table 2:** The association between thrombocytopenia and the occurrence of clinical outcomes.

Clinical outcomes	With TCP (*n* = 936)	Without TCP (*n* = 8595)	*p* value	Adjusted odds ratio (95% CI)^$^	*p* value
	*n* (%)				
Primary outcome: all-cause mortality, bleeding, or transfusion	37 (4.0)	215 (2.5)	0.009	1.30 (0.88–1.93)	0.19
All-cause mortality	16 (1.7)	93 (1.1)	0.09	1.23 (0.67–2.25)	0.50
Bleeding	14 (1.5)	85 (1.0)	0.15		
BARC ≥ 2	12 (1.3)	51 (0.6)	0.014	1.90 (0.98–3.68)	0.06
BARC 1	2 (0.2)	34 (0.4)			
BARC 2	6 (0.6)	21 (0.2)			
BARC 3a	4 (0.4)	16 (0.2)			
BARC 3b	1 (0.1)	9 (0.1)			
BARC 3c	0 (0)	3 (0)			
BARC 5a	1 (0.1)	2 (0)			
Transfusion	18 (1.9)	90 (1.0)	0.016	1.53 (0.88–2.66)	0.13
RBC transfusion	16 (1.7)	79 (0.9)	0.021	1.52 (0.85–2.72)	0.16
Platelet transfusion	1 (0.1)	4 (0.05)	0.99		
Plasma transfusion	4 (0.4)	18 (0.2)	0.34		
Secondary outcome: MACE or CVA	28 (3.0)	212 (2.5)	0.33	1.03 (0.68–1.57)	0.89
MACE	19 (2.0)	123 (1.4)	0.15	1.14 (0.67–1.94)	0.63
Cardiac mortality	13 (1.4)	85 (1.0)	0.25	1.08 (0.56–2.06)	0.82
Myocardial infarction	3 (0.3)	29 (0.3)	1.0		
Target vessel revascularization	3 (0.3)	5 (0.1)	0.042	4.93 (1.13–21.46)	0.03
Stent thrombosis	0 (0)	8 (0.1)	1.0		
Ischemic CVA	9 (1.0)	93 (1.1)	0.73	0.83 (0.41–1.65)	0.59
Hemorrhagic CVA	0 (0)	3 (0)	1.0		
Length of stay (days)	5 (3–7)	4 (3–6)	0.007	0.01 (-0.01–0.02)^&^	0.52

PCI: percutaneous coronary intervention; TCP: thrombocytopenia; CI: confidence interval; BARC: Bleeding Academic Research Consortium; RBC: red blood cell; MACE: major adverse cardiovascular events, including in-hospital cardiac mortality, myocardial infarction, target vessel revascularization, or stent thrombosis; CVA: cerebrovascular accident. ^$^Adjusted for the propensity score in regression models. ^&^*β* coefficient (95% CI) of lg10-transformed length of stay.

**Table 3:** The association between advanced thrombocytopenia (platelet count ≤ 85 × 10^9^/L) and the occurrence of clinical outcomes.

Clinical outcomes	Adjusted odds ratio (95% CI)	*p* value
Primary outcome	1.67 (1.06–2.65)	0.029
All-cause mortality	1.17 (0.56–2.45)	0.68
Bleeding	1.38 (0.67–2.87)	0.384
BARC ≥ 2	2.35 (1.11–5.00)	0.027
Transfusion	2.16 (1.17–4.00)	0.014
Secondary outcome	1.27 (0.76–2.12)	0.366

CI: confidence interval; BARC: Bleeding Academic Research Consortium.

**Table 4:** The association between thrombocytopenia and the occurrence of in-hospital outcomes in the ACS cohort (*n* = 5078).

	ACS cohort (*n* = 5078)				
Clinical outcomes	With TCP (*n* = 497)	Without TCP (*n* = 4581)	*p* value	Adjusted odds ratio (95% CI)^$^	*p* value
	*n* (%)				
Primary outcome: all-cause mortality, bleeding, or transfusion	27 (5.4)	143 (3.1)	0.007	1.33 (0.82–2.16)	0.25
All-cause mortality	13 (2.6)	59 (1.3)	0.017	1.44 (0.71–2.93)	0.31
Bleeding	12 (2.4)	55 (1.2)	0.024	1.71 (0.88–1.71)	0.12
BARC ≥ 2	11 (2.2)	31 (0.7)	0.001	2.56 (1.24–5.44)	0.011
Transfusion	13 (2.6)	62 (1.4)	0.027	1.44 (0.74–2.82)	0.28
RBC transfusion	13 (2.6)	53 (1.6)	0.006	1.74 (0.88–3.44)	0.11
Platelet transfusion	1 (0.2)	2 (0)	0.69		
Plasma transfusion	3 (0.6)	11 (0.2)	0.31		
Secondary outcome: MACE or CVA	19 (3.8)	123 (2.7)	0.14	1.10 (0.65–1.86)	0.73
MACE	15 (3.0)	75 (1.6)	0.027	1.34 (0.71–2.53)	0.36
Cardiac mortality	11 (2.2)	55 (1.2)	0.058	1.37 (0.65–2.90)	0.41
Myocardial infarction	3 (0.6)	18 (0.4)	0.74		
Target vessel revascularization	1 (0.2)	2 (0)	0.69		
Stent thrombosis	0 (0)	4 (0.1)	1.0		
Ischemic CVA	4 (0.8)	53 (1.2)	0.63		
Hemorrhagic CVA	0 (0)	1 (0)	1.0		
Length of stay (days)	5 (3–7)	4 (3–7)	0.017	0.01 (-0.01–0.02)^&^	0.56

ACS: acute coronary syndrome; TCP: thrombocytopenia; CI: confidence interval; MACE: major adverse cardiovascular events, including in-hospital cardiac mortality, myocardial infarction, target vessel revascularization, or stent thrombosis; CVA: cerebrovascular accident; BARC: Bleeding Academic Research Consortium; RBC: red blood cell. ^$^Adjusted for the propensity score in regression models. ^&^*β* coefficient (95% CI) of lg10-transformed length of stay.

## Data Availability

The data underlying the findings of the paper are freely available on request through the authors themselves. Yong He (Department of Cardiology, West China Hospital of Sichuan University, 37 Guo Xue Xiang, Chengdu, Sichuan, 610041, China; e-mail: heyong_huaxi@163.com) should be contacted to request the data.

## Supplementary Materials

Supplementary materials Supplemental figure 1. The association between tirofiban using and the risk of in-hospital outcomes in patients with and without TCP. TCP, thrombocytopenia; BARC, Bleeding Academic Research Consortium; MACE, major adverse cardiovascular events; CVA, cerebrovascular accident. Supplemental Table 1. The occurrence of clinical outcomes among PCI cohort without TCP, and with intermediate or advanced thrombocytopenia. Supplemental Table 2. In-hospital outcomes in CCS patients with and without TCP (n = 4453).
