# Supplementary material for: Impact of Thrombocytopenia on In-Hospital Outcome in Patients Undergoing Percutaneous Coronary Intervention
Source: Cardiovasc Ther. 2021 Jan 13;2021:8836450. doi: 10.1155/2021/8836450 (PMC7817307; doi:10.1155/2021/8836450)
Supplement: Supplementary materials — Supplemental figure 1. The association between tirofiban using and the risk of in-hospital outcomes in patients with and without TCP. TCP, thrombocytopenia; BARC, Bleeding Academic Research Consortium; MACE, major adverse cardiovascular events; CVA, cerebrovascular accident. Supplemental Table 1. The occurrence of clinical outcomes among PCI cohort without TCP, and with intermediate or advanced thrombocytopenia. Supplemental Table 2. In-hospital outcomes in CCS patients with and without TCP (n = 4453). [file 8836450.f1.docx]

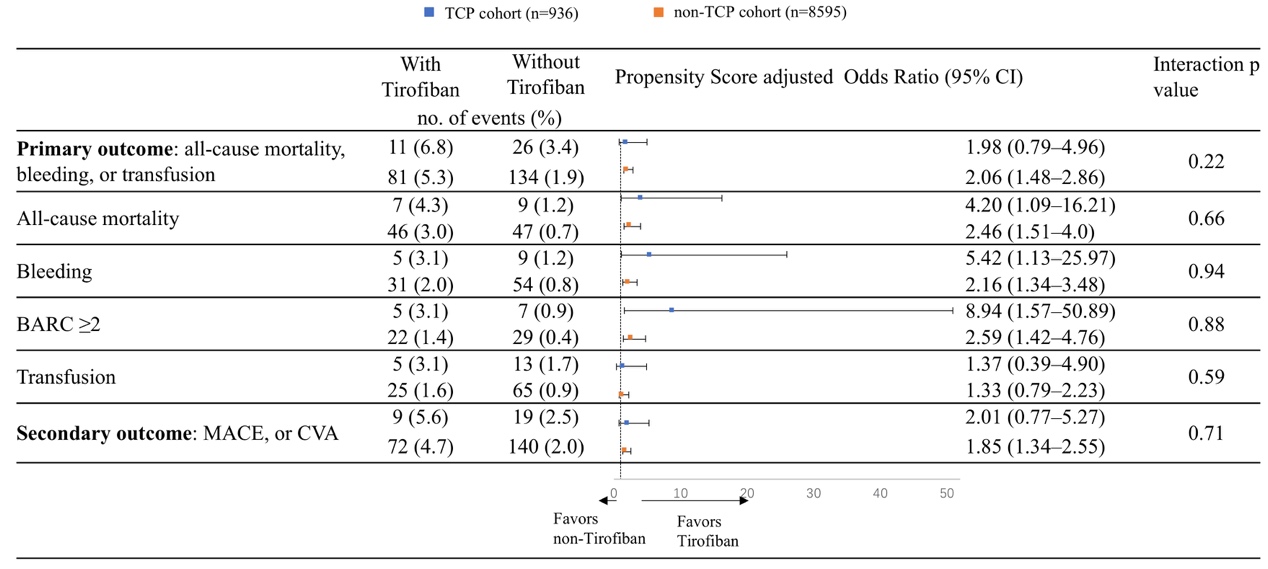


Supplemental figure 1. The association between tirofiban using and the risk of in-hospital outcomes in patients with and without TCP. TCP, thrombocytopenia; BARC, Bleeding Academic Research Consortium; MACE, major adverse cardiovascular events; CVA, cerebrovascular accident.

Supplemental Table 1. The occurrence of clinical outcomes among PCI cohort without TCP, and with intermediate or advanced thrombocytopenia.

| Clinical outcomes | Without TCP  (n = 8595) | With intermediate TCP (n = 463) | With advanced TCP  (n = 473) | p value |
| --- | --- | --- | --- | --- |
| **Primary outcome**, n (%) | 215 (2.5) | 11 (2.4) | 26 (5.5) | **< 0.001** |
| all-cause mortality, n (%) | 93 (1.1) | 6 (1.3) | 10 (2.1) | 0.115 |
| Bleeding, n (%) | 85 (0.99) | 5 (1.1) | 9 (1.9) | 0.161 |
| **BARC ≥2**, n (%) | 51 (0.59) | 3 (0.65) | 9 (1.9) | **0.003** |
| **Transfusion**, n (%) | 90 (1.0) | 5 (1.1) | 13 (2.7) | **0.003** |
| Secondary outcome, n (%) | 212 (2.5) | 10 (2.2) | 18 (3.8) | 0.171 |

PCI, percutaneous coronary intervention; TCP, thrombocytopenia. The chi-square test was performed among the 3 groups. A p value of < 0.017 was assumed to be significant.

Supplemental Table 2. In-hospital outcomes in CCS patients with and without TCP (n = 4453).

| Outcomes | CCS cohort (n=4453) | | | |
| --- | --- | --- | --- | --- |
|  | With TCP  (n=439) | Without TCP  (n=4014) | | *p* Value |
|  | n (%) | | |  |
| **Primary outcome**: all-cause mortality, bleeding, or transfusion | 10 (2.3) | | 72 (1.8) | 0.47 |
| All-cause mortality | 3 (0.7) | | 34 (0.8) | 0.94 |
| **Bleeding** | 2 (0.5) | | 30 (0.7) | 0.70 |
| BARC ≥2 | 1 (0.2) | | 20 (0.5) | 0.68 |
| **Transfusion** | 5 (1.1) | | 28 (0.7) | 0.47 |
| RBC transfusion | 3 (0.7) | | 26 (0.6) | 1.0 |
| Platelet transfusion | 0 (0) | | 2 (0) | 1.0 |
| Plasma transfusion | 1 (0.2) | | 7 (0.2) | 1.0 |
| **Secondary outcome**: MACE, or CVA | 9 (2.1) | | 89 (2.2) | 0.82 |
| MACE | 4 (0.9) | | 48 (1.2) | 0.60 |
| Cardiac mortality | 2 (0.5) | | 30 (0.7) | 0.49 |
| Myocardial infarction | 0 (0) | | 11 (0.3) | 0.55 |
| Target vessel revascularization | 2 (0.5) | | 3 (0.1) | 0.13 |
| Stent thrombosis | 0 (0) | | 4 (0.1) | 1.0 |
| Ischemic CVA | 5 (1.1) | | 40 (1.0) | 0.97 |
| Hemorrhagic CVA | 0 (0) | | 2 (0) | 1.0 |
| Length of stay, days | 5 (3–7) | | 4 (3–6) | 0.16 |

CCS, chronic coronary syndrome; TCP, thrombocytopenia; BARC, Bleeding Academic Research Consortium; RBC, red blood cell; MACE, major adverse cardiovascular events; CVA, cerebrovascular accident. Adjusted odds ratio for the primary outcome was 1.28, 95% CI 0.62 to 2.61, p = 0.51; Adjusted odds ratio for the secondary outcome was 0.92, 95% CI 0.45 to 1.88, p = 0.81.
